# Evaluation of Horn Bud Wound Healing Following Cautery Disbudding of Dairy Calves With and Without the Use of Oxytetracycline Aerosol Spray

**DOI:** 10.3389/fvets.2022.745632

**Published:** 2022-02-24

**Authors:** Rachel Ridgway, Joseph Neary, Andrea Turner, David C. Barrett, Amy Gillespie

**Affiliations:** ^1^Institute of Infection, Veterinary and Ecological Sciences, Livestock and One Health, University of Liverpool, Neston, United Kingdom; ^2^School of Veterinary Science, University of Bristol, Bristol, United Kingdom

**Keywords:** disbudding, dairy calf, wound healing, oxytetracycline, burn wound

## Abstract

Oxytetracycline is commonly applied as a topical agent to burn lesions post cautery disbudding of calves. Judicial use of antibiotics dictates that they should only be used where necessary to reduce the development of resistance in target bacteria. The objective of this study, therefore, was to evaluate the efficacy of topical oxytetracycline spray on wound healing post cautery disbudding of dairy calves over a 6-week period. Dairy calves were disbudded by veterinarians, technicians, or veterinary surgeons, using a standard cautery disbudding protocol. Oxytetracycline spray was randomly applied to the right or left horn bud of each animal (OXY), while the other horn bud received no antibiotic spray (NA). The outcomes measured were wound diameter (WD) and lesion score (LS), either normal healing (NH) or abnormal healing (AH). These assessments were conducted every 14 days following disbudding, until 42 days. A total of 360 animals completed the study. There was a difference in wound diameter and lesion score on day 14 post disbudding between the two groups. Cautery lesions sprayed with oxytetracycline (OXY) were 0.5 ± 0.15 mm smaller than NA lesions (*P* = 0.001), and there were fewer abnormal healing lesions for OXY compared to the NA (2.5 vs. 11%, respectively; *P* ≤ 0.001). There were no differences at day 28 and day 42 post disbudding, and on day 42, 34% of wounds had healed in both groups.

In summary, the authors were unable to demonstrate a difference in healing between the groups using the described methods.

## Introduction

Disbudding dairy calves is a common procedure necessary for modern farm management of cattle to prevent injuries, improve safety, and reduce housing space requirements. There has been much research into the pain and welfare of calves undergoing the procedure (1–4). Adcock and Tucker (3) found disbudding at 3 days old may cause a systemic increase in pain sensitivity compared to disbudding at 35 days old, and Casoni et al. (4) found that 38% of the calves in their study showed chronic sensitization post disbudding, with pain scores significantly higher at 105 days than control animals. Recent literature has also explored potential factors that could affect wound healing, such as the type of hot iron used (5), which found no difference in latency to re-epithelialization between two different types of disbudding iron. There have been three studies to date that monitored how topical applications affected wound healing post cautery disbudding (6–8). Huebner et al. (6) found that an aluminum-based aerosol bandage improved wound healing 3 weeks post procedure compared to no treatment. Stilwell and Laven (7) compared an oxytetracycline aerosol spray with a topical anesthetic and antiseptic gel and suggested that the gel could replace the traditional use of topical antibiotic. Sheil et al. (8) assessed the same topical local anesthetic and antiseptic gel as Stilwell and Laven (7). They found that the product did not adversely affect wound healing and that there was a reduced incidence of abnormal wounds 11–12 days post disbudding in the treated calves compared to a saline placebo. It has not, however, been reported in the literature how topical oxytetracycline aerosol spray used post disbudding would affect wound healing compared with no application of any topical agent.

Oxytetracycline is a broad-spectrum bacteriostatic antimicrobial that is commonly applied as a topical agent to the burn lesion post disbudding. Antimicrobial resistance is a serious issue worldwide for both human and animal health (9). This antimicrobial is being used as prophylaxis, with limited evidence to support widespread prophylactic or therapeutic use of topical antimicrobials (10). Cramer et al. (11) showed the presence of tetracycline in the plasma of cows 7 days after they received topical oxytetracycline for digital dermatitis. The fact that topical oxytetracycline is absorbed systemically could have the potential to impact on the microbiome of the gastrointestinal tract or encourage resistant bacteria to develop.

Although burn wounds are initially considered free of major microbial contamination immediately after a thermal burn, gram positive bacteria, such as staphylococci species, quickly colonize (12). After the first week, the wounds are colonized with mainly gram-negative bacteria, such as *Pseudomonas* spp., as well as gram-positive bacteria and yeast (13). The topical oxytetracycline aerosol used in this study is licensed in the UK to be reapplied every 12 h for 1 to 3 days for sustained activity; however, it is not commonplace to perform a repeat application. Therefore, the objective of this study was to assess the efficacy of a single application of a topical oxytetracycline spray on wound healing post cautery disbudding of dairy calves by evaluating wound healing over a 6-week period.

## Materials and Methods

### Study Population and Study Design

This study was approved by the ethics committee at the University of Bristol (VIN/18/082) and the University of Liverpool (VREC846).

Holstein Friesian dairy calves and dairy cross calves of both sexes from a convenience sample of nine farms that were clients of Leahurst farm animal practice and Langford farm animal practice were enrolled in the study between February 2019 and August 2020. All calves enrolled showed no signs of ill health and were reared as per normal farm practice for each farm, with milk replacer fed twice daily *via* a bucket or bucket and teat. Calves were housed in individual hutches or small group pens on straw bedding at the time of disbudding. Calves of any age were enrolled if they could be disbudded using the disbudding iron only.

Prior to disbudding, a cornual nerve block was administered bilaterally using 2–4 ml procaine hydrochloride 50 mg/ml and adrenaline 0.02 mg/ml (Adrenacaine™, Norbrook) and a subcutaneous injection of meloxicam 0.5 mg/kg (Metacam™, Boehringer Ingelheim) was given. Up to 10 calves were given the cornual block before disbudding to give sufficient time for the local anesthetic to work, and anesthesia was checked with a pin prick.

Calves were disbudded by veterinary surgeons, technicians, or senior veterinary students under the supervision of the technicians or veterinarians. A standard operating procedure was used for cautery disbudding: the area surrounding the horn buds was clipped with scissors if needed, and a butane-fuelled cautery disbudding iron was used to remove the horn bud germinal epithelium including the center of the horn bud. Sutherland et al. (14) found that not removing the central horn bud tissue post disbudding reduced the efficacy from 100% no regrowth of horn at 6 months old with removal to 91% without removal. A Calor-type gas dehorner, 15-mm tip (Agrihealth), was used on eight of the farms, and a Portasol calf dehorner III, 15-mm tip (Portasol^®^), was used on one farm. The principal author directly oversaw the disbudding technique at both veterinary schools.

For the disbudding procedure, the calf was restrained manually in their home pen, and the calves' head was restrained either by a halter or by the disbudding operator. One operator disbudded both horn buds of the calf, and they used their dominant hand to disbud each side. The technique was always the same: place the iron on the bud and hold for several seconds before rolling the iron around the edges of the bud until a deep copper ring had formed and the center could be scooped out.

Operators were blinded to the side that would be sprayed with oxytetracycline spray (Engemycin™ 25 mg/ml, MSD); this was randomly assigned to either the left or right horn bud after disbudding by following the record sheets with the side to spray generated from a random number list in Excel 2016 (Microsoft). The case bud received topical oxytetracycline aerosol (OXY) for 2 s directly on the disbudding site and up to 1 cm around. The control bud did not receive any topical antibiotic (NA) and was protected from any spray by covering while the oxytetracycline aerosol was being applied to the other side. There was no suitable placebo; therefore, the control bud received no spray at all. In the summer months, a fly-repellent cream was applied on the hair around both disbudding sites at the discretion of the veterinary surgeon or technician. Calves were then observed every 14 days over a 6-week period by an assessor.

### Measured Outcomes

**Figure 1:**
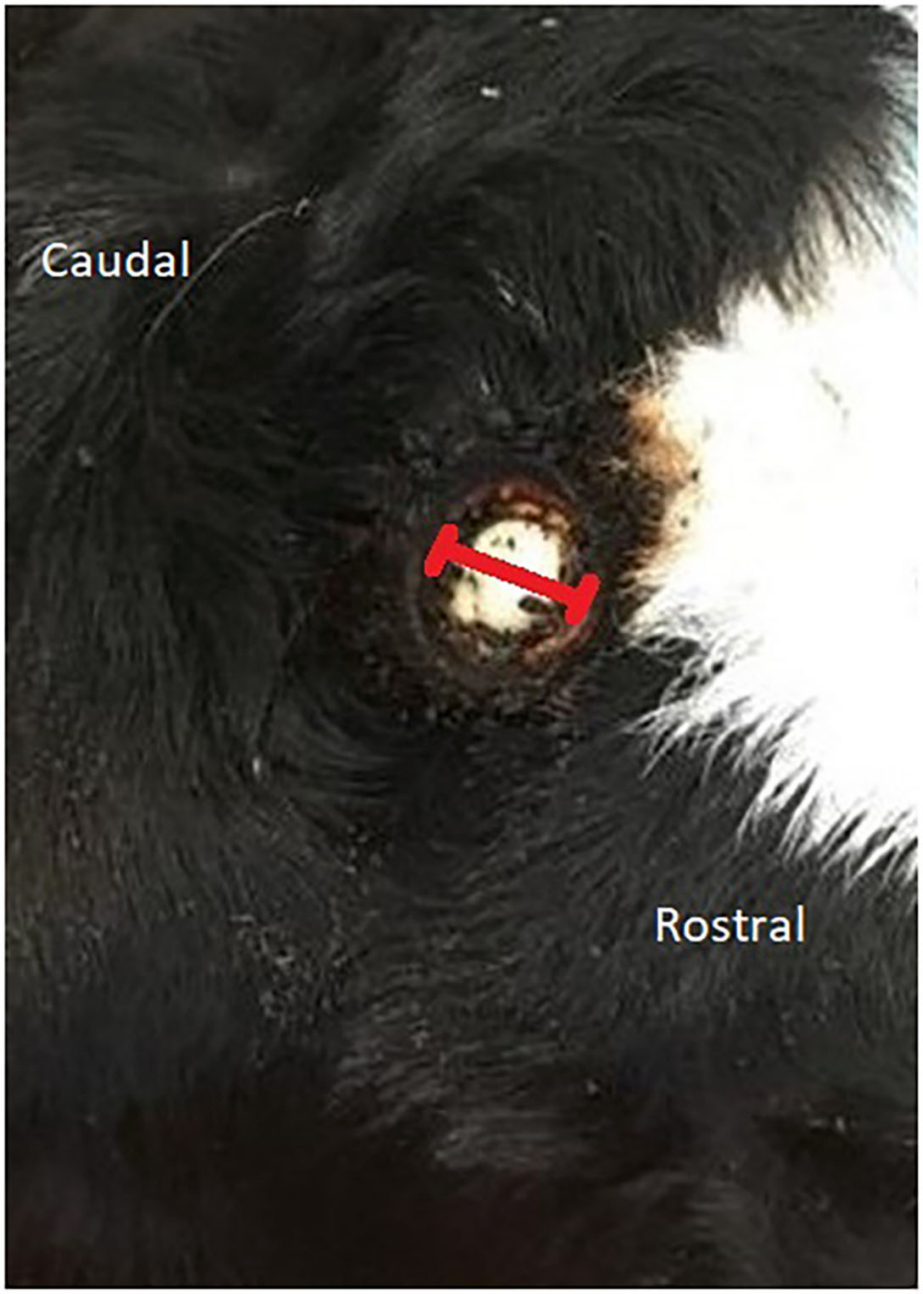
The direction of the measurement of the wound post disbudding.

The wound diameter (WD) was measured to the nearest half a millimeter using a metal ruler across the inner edges of the cautery ring at the widest point in a rostral to caudal direction (Figure 1) and was used as a gauge for healing speed.

**Figure 2:**
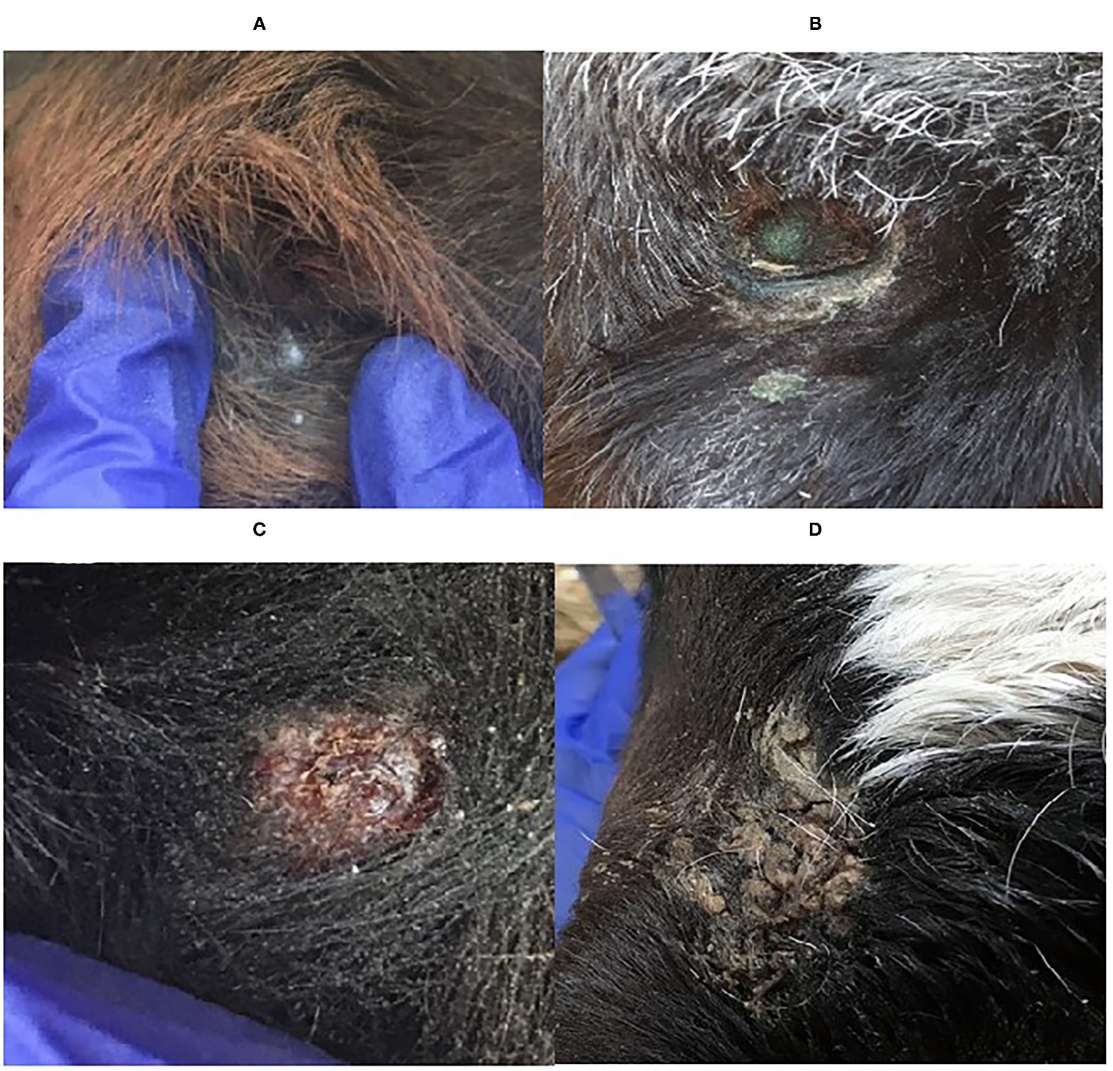
Photographs showing the three different lesion scores used in the study. **(A)** healed (H), **(B)** and **(C)** normal healing (NH), and **(D)** abnormal healing (AH).

A lesion score (LS) system for healing was used based on the normal healing process photographed and documented by Adock and Tucker (3) and the lesion score system by Huebner et al. (6). Abnormal healing was classified as any lesion with a dry or moist purulent discharge. The lesion scores were classified as either healed (H, Figure 2A), normal healing (NH, Figures 2B,C), or abnormal healing (AH, Figure 2D).

WD and LS were determined by assessor (RR and AT) every 14 days during a 6-week follow-up period. Measurements were not taken immediately after disbudding to avoid contamination, which would have biased results favorably toward oxytetracycline use. Initial lesions were, therefore, determined to be ~15 mm in diameter due to the diameter of the disbudding iron. Random allocation of treatment and control on the same subject avoids the potential for bias due to differences in baseline wound diameters. Although it was not possible to blind the assessor from which side was sprayed for the first measurements on day 14, it was possible to blind them at day 28 and day 42 post disbudding, as the blue coloration associated with oxytetracycline spray had disappeared. It was also possible to blind assessors to the WD and LS data from previous weeks as these were recorded separately and were not available for viewing at the next sampling visit.

The following information was also recorded for each calf enrolled: calf identity, which horn bud received oxytetracycline spray (OXY/NA), calf sex, date of birth, farm, date disbudded, and operator.

### Statistical Analyses

A sample size calculation was performed based on a previous study (6) to find a minimum of a 50% reduction of AH in OXY horn buds compared with NA horn buds, assuming calves have a 17% incidence of AH if they are not sprayed. A minimum of 229 calves were necessary to detect a possible reduction of AH with a 95% confidence interval and a power of 80%. Statistics are provided as mean ± SE unless otherwise indicated.

Descriptive analyses were performed in Excel 2016 (Microsoft). Analyses included histograms of WD for the various time points to check for outliers and calculation of summary statistics. Calves with incomplete data were excluded from the analyses. Data were then imported into STATA 14 (Statacorp, USA) for further analyses.

First, linear regression analyses were performed for each time point (days 14, 28, and 42) on the paired difference (OXY–NA) wound diameter (WD) with farm included as a fixed effect. Likelihood ratio tests were performed to test the statistical significance of farm. Model outcomes are presented as marginal means.

Mixed-effect logistic regression analyses of lesion healing were performed for days 14 and 28 post disbudding with clustering by farm and calf accounted for as random effects. The dichotomous outcome consisted of H and NH (0) vs. AH (1). Logistic regression analyses were not performed at day 42 due to model instability as there were only two AH wounds. Bonferroni correction was used to achieve a family-wise error rate of 0.05. As five models were performed, this meant that a probability value of <0.01 was considered sufficient to reject the null hypothesis of no difference. Variance estimate of the farm-level random effect was <0.0001, indicating some model instability due to the low number of farms included in the analysis.

## Results

### Descriptive Results

In total, 474 calves were enrolled, and 114 calves were excluded because of incomplete data sets due to either being sold or inability to visit the farm, leaving 360 calves in the study. Calves from nine farms were enrolled; eight farms were dairy units, and one was a calf-rearing unit (Table 1). One hundred and seventy-nine calves had the left side sprayed and 181 calves had the right side sprayed with topical oxytetracycline antibiotic.

**Table 1:** Number of calves enrolled from each farm.

**Farm number**	**Number of calves enrolled**
1	11
2	15
3	2
4	51
5	23
6	88
7	54
8	56
9	60

The mean age of calves disbudded was 41 ± 16.4 days; 337 were females, 21 were males, and two calves had no data on sex. One hundred and sixty-three calves were disbudded by a veterinary student compared to 197 calves disbudded by a technician/veterinary surgeon, and there were six different breeds/dairy crosses. Ninety-seven calves were disbudded in the defined fly season (June, July, and August), and 263 were disbudded during the rest of the year. One investigator (RR) made 891/1,083 assessments of WD and LS, and the remaining 192 assessments were made by another investigator (AT).

### Wound Diameter Results

On day 14 post disbudding, on average across all farms, the wound diameter for OXY was 0.5 mm smaller than NA (*P* = 0.001) (Table 2). Farm had no effect on whether there was a difference between OXY and NA (*P* = 0.23). On day 28 post disbudding, on average across all farms, the wound diameter for OXY was no different in size to NA (*P* = 0.89), and farm had no effect on whether there was a difference between OXY and NA (*P* = 0.35). On day 42 post disbudding, on average across all farms, WD for OXY was no different in size to NA (*P* = 0.69). Farm tended to influence whether there was a difference between OXY or NA (*P* = 0.03).

**Table 2:** Linear regression analysis on the paired difference wound diameter (WD) with farm as fixed effect.

	**Marginal mean WD, mm**	**SE**	**Range, mm**	**Marginal mean WD difference (OXY-NA), mm**	**95% CI, mm**	***P*-value**
Day 14 post disbudding						
OXY^a^	14.82	0.12	10–30	−0.5	−0.77 to −0.20	0.001
NA^b^	15.31	0.13	10–30			
Day 28 post disbudding						
OXY	11.08	0.20	2–30	0.03	−0.36 to 0.41	0.89
NA	11.05	0.24	0–30			
Day 42 post disbudding						
OXY	4.20	0.19	0–22	0.07	−0.42 to 0.27	0.69
NA	4.23	0.20	0–20			

a *OXY disbudding sites were sprayed with topical oxytetracycline aerosol after disbudding*.

b *NA disbudding sites received no spray after disbudding*.

### Lesion Score Results

**Figure 3:**
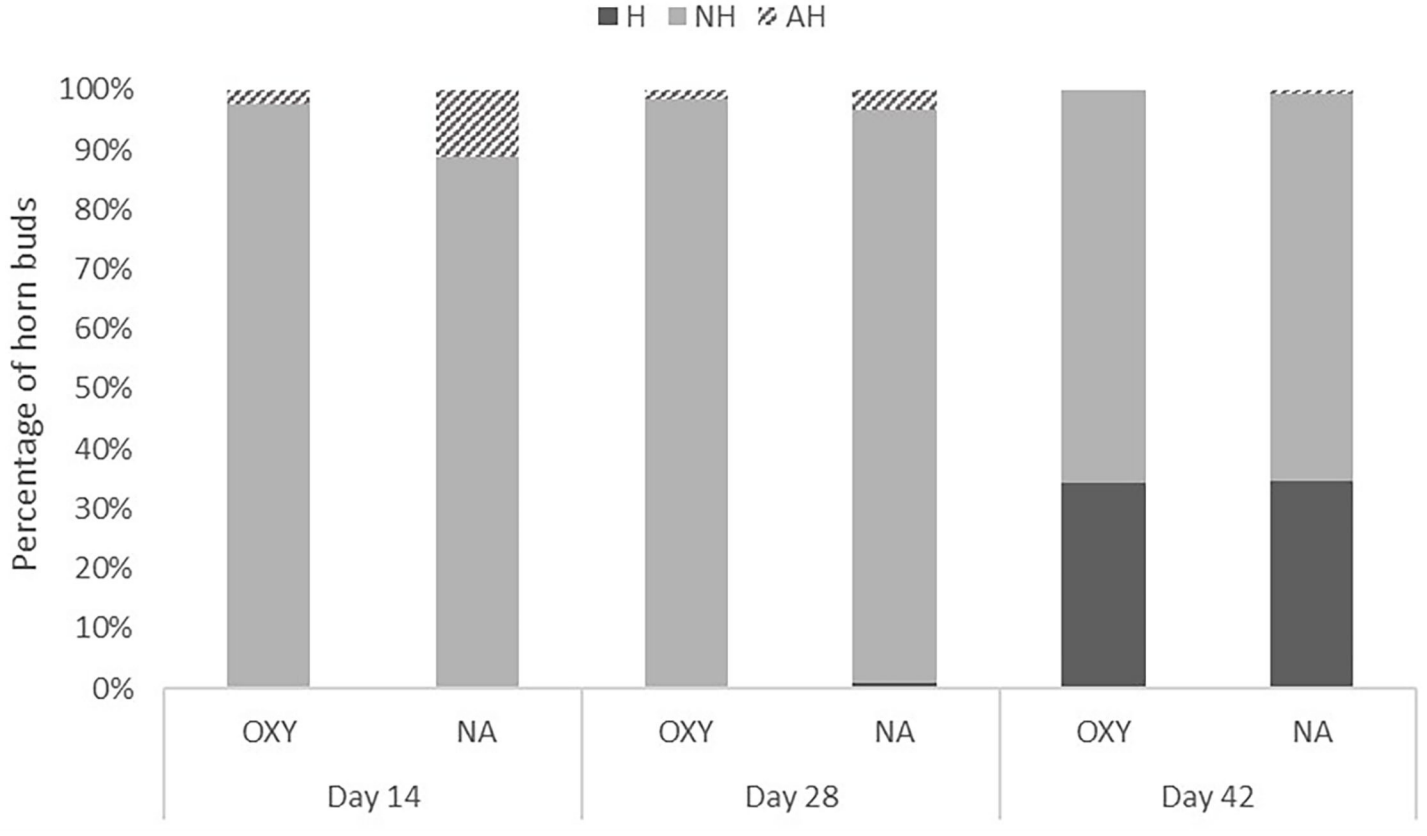
Prevalence of lesion scores (LS) by study groups (OXY vs. NA). H, healed; NH, normal healing; AH, abnormal healing at 14, 28, and 42 days post disbudding.

Figure 3 shows that at 14 days post disbudding, 97.5% (351) of OXY were NH compared to 88.9% (320) of NA; 2.5% (9) of OXY were AH compared to 11.1% (40) of NA. At 28 days post disbudding, no OXY were H compared to 0.8% (3) of NA; 98.3% (354) and 95.9% (345) OXY and NA were NH, respectively; 1.7% (6) of OXY were AH compared to 3% (12) NA. At 42 days post disbudding, 34.4% (124) and 34.7% (125) of OXY and NA were H, respectively; 65.5% (236) and 64.7% (233) of OXY and NA had NH, respectively. No OXY had AH compared with 0.6% (2) NA.

Calves were more likely to have AH in the NA group than the OXY group at day 14 (*P* ≤ 0.001) but not at day 28 (*P* = 0.16, Table 3). Only two calves had AH at day 42; both calves were in the NA group.

**Table 3:** Mixed-effect logistic regression analyses for lesion healing with farm and calf accounted as random effects.

	**Odds of AH**	**95% CI**	***P*-value**
Day 14 post disbudding			
NA^b^	1.0	0.07 to 0.39	<0.001
OXY^a^	0.16		
Day 28 post disbudding			
NA	1.0	0.18 to 1.32	0.16
OXY	0.49		

a *OXY disbudding sites were sprayed with topical oxytetracycline aerosol after disbudding*.

b *NA disbudding sites received no spray after disbudding*.

## Discussion

The aim of this study was to evaluate wound healing following cautery disbudding of dairy calves with and without the use of oxytetracycline aerosol. There have only been three studies comparing healing of disbudding wounds and topical treatments, of which none compared oxytetracycline aerosol to no spray. An aluminum barrier spray was assessed against no spray (6); oxytetracycline aerosol was compared with Tri-solfen™ gel (7), and Tri-solfen™ gel was assessed against no gel application (8). Our results show a higher prevalence of abnormal healing in the NA group at day 14 post disbudding, with significant differences for both WD and LS; however, no significant difference between the two groups was found at 28 and 42 days post disbudding, and both groups had 34% of horn buds healed by 42 days. Although healing was not complete for all horn buds by 42 days, as there were no differences in WD and LS beyond 14 days, further follow-up would be unlikely to be relevant. None of the AH lesions were considered severe enough to warrant further treatment, and so this was not a factor affecting the results.

Huebner et al. (6) suggested that a topical aluminum aerosol bandage improved wound healing over the 3-week follow-up. However, they assumed that a raised scab was abnormal healing, whereas we classified it as normal healing based on the system by Adcock and Tucker (3). They only found significant differences between the ALU group and the control group in week three, which was when the study ended. They did state that if they had continued monitoring for a longer period, the effects of the aluminum spray may have become more evident. This is relevant as the average healing time for disbudding wounds has been found to be 63 days by Adcock and Tucker (3) and 54 days by Adcock et al. (5).

Stilwell and Laven (7) recently compared topical oxytetracycline and a viscous gel product containing lidocaine, bupivacaine, adrenaline, and cetrimide (Tri-solfen™, Dechra), which has recently been licensed in Australia and New Zealand for use on disbudding wounds. The authors found a greater prevalence of granulation tissue and less crusting in disbudding wounds treated with the topical gel compared to oxytetracycline. Their study suggests that treatment with the gel could replace the traditional use of topical antibiotic after disbudding. They did state that this was a small-scale study, and one of the limitations was that the animals were only evaluated once, 7 days post disbudding. Sheil et al. (8) investigated the safety and impact on wound healing of Tri-solfen™ (Dechra) under field conditions and found no clinically significant differences between the product and the placebo over a period of 34 days. If this product were to become licensed in the UK, it has the potential to become an alternative to using oxytetracycline as well as providing additional pain relief.

The difference in WD at 14 days of 0.5 mm, although statistically significant, may not be so relevant in the clinical situation. A limitation of the study was that an initial measurement was not taken. This decision was made with calf welfare in mind and that the wound could have been contaminated if it was touched straight after disbudding. Also, the assessors may not have been accurate down to half a millimeter, as although measurements were repeated if the calf moved, the repeated measurements were not recorded and then averaged. A more accurate method would have been to take a photograph of each wound with a tape measure in the photo and then measure WD and to have performed this at disbudding.

Having four and a half more times abnormal lesions in the NA group than the OXY group at day 14 (40 vs. 9, respectively) could be more relevant in a clinical situation; however, that is still only 11% (40/360) of horn buds with abnormal healing with no application of antibiotic. Although WD is objective and the lesion scores were quite distinct with photographs to compare the lesions to, the 14-day post disbudding measurements could not be blinded, and a spurious result could not be completely ruled out. This is a limitation of the study, in which a suitable placebo could not be formulated containing all the products except for the antibiotic.

The aerosol used in this study also contains patent blue v (synthetic dye), polysorbate 80 (a non-ionic surfactant and emulsifier), isopropyl alcohol, and a mixture of hydrocarbons on a butane basis with denaturant. The alcohol in it could have contributed to drying out the wound, producing a smaller WD and making it more likely to have a normal LS.

A strength of our study design with the random allocation of treatment and control on the same subject is that it avoids the potential for bias due to differences that were extraneous to the study question, such as the disbudding operator or the equipment used. In a recent study, Adcock et al. (5) did not find a difference in healing between two different disbudding irons.

The volume of oxytetracycline spray used for each individual calf is small, estimated at 125 mg/calf (25 mg/ml, sprayed for 2 s is ~2.5 ml/bud). However, this accumulates when applied to every calf born on the farm with horns. Reducing the use of antibiotic sprays in farm procedures may be an important tool in the fight against worldwide bacterial resistance (7), as topical antibiotic agents change the microbiologic environment of the skin, predisposing to potentially resistant pathogens, and so, antibiotics should be used judiciously (15).

It is not surprising that there was no difference found between the groups in the long term because of the 12-h duration of the oxytetracycline aerosol, further supporting the assertion that using the antibiotic spray may not be beneficial.

## Conclusions

The authors were unable to demonstrate a difference in healing between the groups using the described methods.

## Data Availability Statement

The raw data supporting the conclusions of this article will be made available by the authors, without undue reservation.

## Ethics Statement

The animal study was reviewed and approved by the University of Liverpool and the University of Bristol. Written informed consent was obtained from the owners for the participation of their animals in this study.

## Author Contributions

All authors listed have made a substantial, direct, and intellectual contribution to the work and approved it for publication.

## Funding

Gold open access funding via University of Liverpool library PO number 100679356 - needs to be on the invoice VAT number GB 673 5988 75.

## Conflict of Interest

The authors declare that the research was conducted in the absence of any commercial or financial relationships that could be construed as a potential conflict of interest.

## Publisher's Note

All claims expressed in this article are solely those of the authors and do not necessarily represent those of their affiliated organizations, or those of the publisher, the editors and the reviewers. Any product that may be evaluated in this article, or claim that may be made by its manufacturer, is not guaranteed or endorsed by the publisher.
